# Generative AI: Potential and Pitfalls in Academic Publishing

**DOI:** 10.1097/PG9.0000000000000387

**Published:** 2023-11-08

**Authors:** Jason A. Silverman, Sabina A. Ali, Anna Rybak, Johannes B. van Goudoever, Neal S. Leleiko

**Affiliations:** From the * Department of Pediatrics, University of Alberta Edmonton, AB, Canada; † Clinical Professor of Pediatrics, University of California San Francisco; ‡ Department of Gastroenterology, Great Ormond Street Hospital London, UK; § Amsterdam UMC, University of Amsterdam, Vrije Universiteit, Emma Children’s Hospital Amsterdam, The Netherland; ∥ Professor of Pediatrics, Columbia University Irving Medical Center New York, NY.

Generative artificial intelligence (AI) has attracted enormous attention since the release of ChatGPT (1) in late 2022. Other generative AI chatbots used to generate text (including Bard, Claude, and CoPilot (2–4)) and images (including Dall-E, Midjourney, and Stable Diffusion (5–7)) have likewise seen a remarkable explosion in development and uptake. While ChatGPT has been promoted for its potential to assist users in efficiently and easily creating text to serve a wide range of purposes, this editorial focuses on the journal’s opinion on the use of generative AI technology in creating articles submitted to this journal.

ChatGPT utilizes a technology known as a generative pretrained transformer large language model (8). This is a form of machine learning in which very large language-based data sets are used to train computers to comprehend natural language. While natural language processing is not new, ChatGPT is currently unique in its ability to not only understand queries and information to which it has access, but also to generate new, comprehensive, and fluent language-based content. Models, such as ChatGPT, which generates language, and Dall-E, Midjourney, or Stable Diffusion, which all generate images, are collectively referred to as “generative AI”, and can be seen as marking a dramatic shift in both the capabilities and widespread access to AI technology.

Since its release on November 30, 2022, ChatGPT achieved the most rapid adoption of a consumer software application in history by amassing over 100 million users by January 2023 (9). Users have leveraged ChatGPT to write software, song lyrics, stories, poems, and letters. With its ability to recall previous prompts within a conversation, users can fine-tune its responses to modify the content or tone of the content it generates. The underlying model used to generate content is continuously improved through feedback from users. While users easily identified flaws in the responses provided by the earlier GPT-3 version, the model has iterated quickly. GPT-4, OpenAI’s latest effort in scaling up deep learning, has been described by its creators to exhibit “human-level performance on various professional and academic benchmarks” (10). It has demonstrated impressive abilities in passing standardized examinations including the Law School Admission Test, Scholastic Aptitude Test, a unified bar exam, and even the United States Medical Licensing Exam (10).

ChatGPT, and other large language model-based applications, use large data sets consisting of text available on the web. Sources may include articles, books, web-based advertising, and social media posts. ChatGPT offers several exciting and desirable potential benefits, including its potential to make completing written articles more quickly as well as completing literature review summaries. With its ability to help users write in English fluently, it has been touted as a way to help make academic publishing more equitable and diverse (11). The time saved in summarizing data and generating articles could help researchers publish their studies faster, yielding more time to work on new experimental designs, grant applications, and more. This could significantly accelerate innovation and potentially lead to breakthroughs across many disciplines. We think this technology has enormous potential; however, important risks and limitations still exist that must also be acknowledged.

Using ChatGPT or other language models may lead to inaccuracies, biases, and unintended plagiarism. It has been repeatedly shown that these models may create well-written text that has little relationship to reality in a phenomenon sometimes referred to as “hallucinations” (12,13). In particular, ChatGPT may generate text complete with appropriate-sounding but completely fictional citations included (14). It is obvious that articles containing factual inaccuracies, invented citations, and plagiarized content do not meet the editorial standards of our journals.

Based on a review of the current capabilities and limitations of available generative AI software, we require our contributors to follow responsible and transparent practices and policies. It is necessary that authors ensure that references cited state what is attributed to them and that the overall written document is logical and consistent with the actual findings reported in the submission. In line with the International Committee of Medical Journal Editors, we expect each submission to reflect the expertise of the author or authors, who are all ultimately responsible for every word in their submission (15). As such, generative AI software, including ChatGPT and others, may not be listed as a coauthor on any submitted article. Authors must clearly state in their article the extent to which AI technologies were used in data analysis, literature review, and article preparation. This will aid reviewers and editors in checking potential biases, inaccuracies, and improper source attribution while providing readers with a transparent view of how the article was created. We ask that authors (and other interested parties) state in their letter of submission the extent to which AI was used as well as other related information they deem pertinent. This information will help ensure the integrity of the editorial review and publication process and will serve as a learning tool for the editors.

We all have a great deal to learn about generative AI and its place in academic publishing. We need to evolve together along with the technical capabilities of AI. We expect to modify our editorial policies and instructions for authors often in the coming months and years as these tools, and our understanding of their impacts and best uses continue to develop.
